# Supplementary material for: Neurodevelopmental delay and associated factors among preterm infants aged 6 to 24 months adjusted gestation age in two urban hospitals in Uganda
Source: PLOS Glob Public Health. 2025 May 29;5(5):e0004361. doi: 10.1371/journal.pgph.0004361 (PMC12121762; doi:10.1371/journal.pgph.0004361)
Supplement: Attachment — Submitted filename: Response to reviewers.docx [file pgph.0004361.s004.docx]

**Dear Editor,**

We are extremely grateful for the opportunity to submit a revised version of our manuscript titled " Neurodevelopmental Delay and Associated Factors among Preterm Infants Aged 6 to 24 months Adjusted Gestation Age in Two Urban Hospitals in Uganda” to PLOS Global Public Health Journal to be considered for publication.

We sincerely appreciate and value the time the editor and the reviewers have invested in reviewing this manuscript and providing such great feedback to us. We have made changes to the manuscript in regards to the recommendations provided by the reviewers. We have put the changes in red so that they are easily seen.

In regards to the journal requirements, we have provided the figure files in the recommended format, provided a full list of legends for supporting information files after the references list, provided all the raw data used for the study and ensured that our reference list is complete.

Here are the responses to the reviewers’ comments.

**Comments from Reviewer 1;**

**Comments about the abstract;**

**Comment;** *For clarity and smooth reading, change
"This study aimed at determining...." to "This study aimed to determine..."*

**Response;** Thank you so much for this correction, we have rectified it as recommended.

**Comment***; "Factors associated with NDD included microcephaly..." → Consider rewording for clarity, e.g., "Significant factors associated with NDD included microcephaly, severe acute malnutrition (SAM), and incomplete immunization."
"Families with more than 5 children" → "Large family size (≥5 children)" might be more concise.*

**Response;** Thank you so much for those recommendations, we have made those changes as advised.

**Comment;** *Statistic: The p-values are presented inconsistently. Some with three decimal places, others with two. Consider formatting them uniformly (e.g., P = 0.002 for all)*

**Response;** Thank you so much for this observation. We have ensured that all P-values are presented consistently with three decimal points.

**Comment;** *Conclusion: Instead of "The 13.6% prevalence of NDD is noteworthy...", consider "NDD affects 13.6% of preterm infants, with social and fine motor delays being most prevalent."*

**Response;** Thank you so much for this correction, we have written the conclusion as per your recommendation.

**Comments on the Introduction**

**Comment;** *The citation "1,2" immediately following the statistic on preterm births could be more specific. For example: "According to the World Health Organization, approximately 15 million preterm births occur annually, with 60% of these occurring in Asia and sub-Saharan Africa." This would clarify the source of the statistic and avoid confusion, especially if the citations are to be included in the references section.*

**Response;** We are in agreement with you. Thank you so much for this recommendation.

**Comment;** *You mentioned that the "7% of preterm infants" and "developmental delay has been shown to increase with decreasing gestational age". You could merge these ideas by saying: "Developmental delay increases with decreasing gestational age, with an estimated 7% of preterm infants globally surviving with neurodevelopmental impairments”*

**Response;** This is such a wonderful recommendation. We have merged the two sentences.

**Comment;** *Consider revising this sentence for better flow: "Prematurity increases the risks for morbidities such as respiratory distress syndrome, sepsis, necrotizing enterocolitis, intraventricular hemorrhage, seizures, hypoxic ischemic encephalopathy, jaundice and kernicterus." You could rephrase it as: "Prematurity increases the risk for morbidities such as respiratory distress syndrome, sepsis, necrotizing enterocolitis, intraventricular hemorrhage, seizures, hypoxic ischemic encephalopathy, jaundice, and kernicterus."*

**Response;** Thank you so much for the guidance, we have rephrased the sentence as advised.

**Comments on the discussion and conclusion**

**Comment;** *Breaking down some of the more complex information into bullet points or subheadings (e.g., Key Risk Factors, Healthcare Infrastructure, Social Determinants) would improve readability and help the reader navigate the discussion more easily*

**Response;** Thank you so much for such an important observation. We have included two subheadings in the discussion; Healthcare Infrastructure and Prevalence of NDD, Key factors associated with NDD among preterm infants.

**Comment;** *While the study compares findings from different regions, it could be helpful to explicitly state at the beginning of the section the main differences between the urban hospital setting in this study and the rural Iganga setting. This could help the reader immediately grasp the key contextual differences before diving into the more technical discussions****................***

**Response;** This is such an important observation and thank you so much for the guidance on how to go about it. We have written a paragraph at the beginning of the discussion section explaining the differences between the two settings.

**Comment;** *The discussion would benefit from specific statistical data or quantitative results from the study to back up claims made in the discussion. For example, when mentioning the increased prevalence of NDD in infants with neonatal sepsis or hypoglycemia, referencing exact percentages or relative risks would make the argument stronger and more grounded in the data*

**Response;** Thank you so much for this comment. We have included the specific statistical data as recommended.

**Comment;** *The conclusion summarizes the findings well, but it could be more impactful by focusing on future research directions such as exploring how early interventions could reduce NDD.*

**Response;** We thank you for this recommendation. We have added a sentence to the conclusion in regards to your recommendation.

**Comment;** *There was no report on whether this occurred in first-time mothers or not, child with a sibling that was full-term or not.*

**Response;** Thank you for this comment. There was no report on these parameters/variables as they were not part of the study objectives and as such data to this effect was neither collected nor analyzed.

**Comments on the references;**

**Comment;** *Ensure references are cited properly with correct DOIs and uniform although.
Check citation 25*

**Response;** We apologize for misquoting that reference, it is supposed to be reference 26, and we have rectified this in the manuscript. We have also ensured that all the other references are uniform and cited properly with correct DOIs as advised.

**Other comments**

**Comment;** *Minor grammar check to ensure repetitions.*

**Response;** We have ensured that grammar in the manuscript is appropriate

**Comments from reviewer 2;**

**Comment;** *194, 197: If this question was asked as per 197, last item in the table (sometimes, rarely or never), I would report it as “perceived support” from others given the very subjective nature of responses. Furthermore, the construct of social support in the context of child care is very variable across settings and I would couch the discussion of findings on this parameter with the deeper explanation of context of the extended family support system and expectations in the setting/ location where this study was conducted so that readers could discern how applicable this would be in their context.*

**Response;** Thank you so much for such pertinent observations. We have changed the question as advised. We have also added a deeper explanation in regards to the same, and two more references (16 and 17) to further enrich the discussion.

**Comment;** *The Iganga study looked at a cohort of babies born prematurely at a rural hospital while this study was among preterm babies attending a follow up clinic in an urban hospital. Delve into the differences conferred by the two approaches and how this could account for (or not) the differences****.*** *Also delve into the inherent selection bias and limitations given this study was done in a follow up clinic and largely in a private urban hospital to help readers to give readers a sense of the internal and external validity of the findings respectively.*

**Response;** Thank you so much for this comment. We have explained the differences between the urban hospitals where this study was carried out and the rural hospital where the Iganga study was conducted and how this may explain the results obtained in this study. This is in the first three paragraphs in the discussion section, from line 207-224.

We are happy to respond to any further questions and comments you may have. We look forward to having our manuscript published in your prestigious journal. Thank you so much for your time.

Sincerely,

Robert Sebunya.
